# Intrauterine growth‐restricted pregnant rats, from placental ischemic dams, display preeclamptic‐like symptoms: A new rat model of preeclampsia

**DOI:** 10.14814/phy2.70112

**Published:** 2024-10-31

**Authors:** Jonna Smith, Madison Powell, Whitney Cromartie, Savanna Smith, Kylie Jones, Angie Castillo, Jordan Shaw, Joseph Editone, Ahfiya Howard, Robert Tatum, Alex Smith, Brandon Fisher, George W. Booz, Mark Cunningham

**Affiliations:** ^1^ Department of Physiology and Anatomy University of North Texas Health Science Center Fort Worth Texas USA; ^2^ School of Social Work Texas A & M University‐Commerce Commerce Texas USA; ^3^ Department of Pharmacology & Toxicology University of Mississippi Medical Center Jackson Mississippi USA

**Keywords:** hypertension, intrauterine growth restriction (IUGR), nitric oxide, oxidative stress, preeclampsia

## Abstract

Preeclampsia (PE) is characterized by de novo hypertension (HTN) and is often associated with intrauterine growth restriction (IUGR). Hallmarks of PE are placental ischemia, decreased nitric oxide (NO) bioavailability, oxidative stress (OS), and organ damage in the kidneys and brain. This study aims to characterize a new model of PE using pregnant IUGR rats from hypertensive placental ischemic dams. It is hypothesized that pregnant IUGR rats from hypertensive placental ischemic dams will have elevated blood pressure (BP), OS, and organ damage. In this study, pregnant rats are divided into two groups: normal pregnant (NP) and hypertensive placental ischemic dams (RUPP). Offspring from NP and RUPP dams were mated at 10 weeks of age to generate pregnant IUGR (IUGR Preg) and pregnant control (CON Preg) rats. BP and other markers of PE were evaluated during late gestation. Pregnant IUGR rats had elevated BP and systemic OS. The maternal body weight of pregnant IUGR rats and their pups' weights were decreased, while the brains were enlarged with elevated OS. In summary, pregnant IUGR rats, born from hypertensive placental ischemic dams, have HTN and increased systemic and brain OS, with larger brain sizes and smaller pups. Furthermore, this study shows that pregnant IUGR rats exhibit a preeclamptic‐like phenotype, suggesting a new epigenetic model of PE.

## 1 INTRODUCTION

Preeclampsia (PE), a pregnancy complication syndrome primarily occurring during the third trimester, is generally defined by new‐onset hypertension (HTN) with one or more of the following conditions: proteinuria, end organ dysfunction, and/or uteroplacental dysfunction (Cunningham Jr. & LaMarca, 2018; Magee et al., 2022). Organ damage and dysfunction in PE patients is commonly presented as proteinuria, thrombocytopenia, increased liver enzymes, acute kidney injury, pulmonary edema, and neurological complications. These neurological complications consist of cerebrovascular dysfunction, altered mental status, new‐onset headaches that are unresponsive to medication, and impaired vision (Amaral et al., 2017; Karrar & Hong, 2023; Magee et al., 2022; *Obstetrics and Gynecology*, 2020; Sibai & Stella, 2009). Furthermore, daughters of women who had PE during pregnancy are twice as likely to develop PE compared with women from normal pregnancies (Sherf et al., 2019; Skjaerven et al., 2005). PE presents a wide variety of manifestations, timing, and severity (Smith et al., 2021). PE causes end organ damage, especially in the kidneys and brain, of both the mother and fetus (Cunningham Jr. & LaMarca, 2018). PE is a major cause of maternal and fetal morbidity, mortality, intrauterine growth restriction (IUGR), and preterm birth (Amaral et al., 2017; Ashraf et al., 2021; Goldenberg et al., 2008; *Obstetrics and Gynecology*, 2019), which affects ~5%–10% of all births in the United States each year (Cunningham Jr. & LaMarca, 2018).

IUGR, which affects ~10% of all pregnancies, is commonly caused by a reduction in placental blood flow, causing a reduction in oxygen and nutrient delivery to the fetus. This usually results in restricted fetal growth and the delivery of small‐for‐gestational‐age babies (LaMarca et al., 2016). PE affects both the mother and the fetus before, during, and after birth. Furthermore, PE increases the risk of cardiovascular disease, cerebrovascular disease, and renal disease for both mother and child later in life (Andraweera et al., 2021; Ashraf et al., 2021; Cunningham Jr. & LaMarca, 2018).

The origins and etiology of PE are not known, and there is no cure for the disease, except for delivery of the fetoplacental unit (Amaral et al., 2017). The pathophysiology of PE, in both clinical and animal studies, is associated with placental ischemia (Amaral et al., 2017; Jena et al., 2020; Jung et al., 2022; LaMarca et al., 2016; Qu & Khalil, 2020), decreased nitric oxide (NO) bioavailability, increased oxidative stress (OS), proinflammatory factors, and antiangiogenic factors (such as soluble FMS‐like tyrosine‐1), along with genetic predisposition (Amaral et al., 2017; Campbell et al., 2018; LaMarca et al., 2016; Vaka et al., 2021). To examine the pathology of PE and to derive new therapies against the development of PE, several rodent models have been created through pharmacological, surgical, and/or genetically induced alterations (Bakrania et al., 2022; Campbell et al., 2018; Gatford et al., 2020; Granger et al., 2006; LaMarca et al., 2016; Taylor & George, 2022). These models have pushed the field forward and provided significant insight into the pathophysiology of PE. However, these models are limited, because rodents, like many other animal species, do not naturally develop PE and require direct outside interventions. Although these models provide a wealth of knowledge about PE, none of these models occur without surgical, pharmacological, and/or genetic intervention. Our model serves as a different model of PE that does not directly require these interventions, while still showing the symptoms of PE. Our model is radically different because it will evaluate some of the symptoms of PE in moms that were exposed to placental ischemia during their fetal development before birth.

Few studies have examined pregnant rodents from second‐generation PE pregnancies, despite their increased risk of PE during pregnancy (Cnattingius et al., 2004; Esplin et al., 2001; Galaviz‐Hernandez et al., 2018; Gallo, Tran, Moritz, Jefferies, & Wlodek, 2012; Gallo, Tran, Moritz, Mazzuca, et al., 2012; Paauw et al., 2017; Zetterstrom et al., 2007). Therefore, in this study, we want to explore the pregnancy phenotype of rat offspring born from PE dams. We hypothesize that pregnant IUGR rats born from hypertensive placental ischemic dams will have HTN, reduced NO bioavailability, and elevated OS. The objective of this study is to characterize this new model of PE with adult pregnant IUGR rats from hypertensive placental ischemic dams.

## 2 METHODS

### 2.1 Animals

Timed‐pregnant Sprague Dawley rats were purchased from Envigo (Indianapolis, IL) to produce offspring (both control and IUGR), which were mated and evaluated in this study. The animal protocols and handling methods were approved by Institutional Animal Care and Use Committee (IACUC) at the University of Mississippi Medical Center, #1543, where we conducted our animal experiments. The rats were housed in temperature‐regulated rooms with 12‐h light–dark cycles. Food and water were administered ad libitum to all rats. All pregnant rats were allowed to give birth naturally and were weaned for 3 weeks. Animal experiments were conducted under the guidelines from the National Institute of Health (NIH) for the use and care of animals. The Western blots and ELISAs were performed at the University of North Texas Health Science Center.

### 2.2 Pregnant IUGR rats from placental ischemic hypertensive dams

All IUGR rats were derived from the placental ischemic hypertensive reduced uterine perfusion pressure (RUPP) model of PE. Note, that RUPP model is a commonly used surgical model of PE to generate IUGR offspring, which experience placental ischemia and are born with low birth weights (Ashraf et al., 2021; Elfarra et al., 2017; LaMarca et al., 2016). Our P1 preeclamptic pregnant dams were randomly separated into two groups: normal pregnant (NP) and RUPP groups. On gestational day 14, RUPP dams received the RUPP surgery, as described by us and others (Amaral et al., 2017; Elfarra et al., 2017; Granger et al., 2006; Jung et al., 2022; LaMarca et al., 2016; McClements et al., 2022; Qu & Khalil, 2020; Vaka et al., 2018). No surgical/sham procedures were performed on the NP dams, as previous studies from our laboratories and others showed no differences in sham versus NP control rats (Amaral, Cornelius, et al., 2015; Morton et al., 2019). Both groups were allowed to progress through their pregnancy and deliver naturally.

**FIGURE 1:**
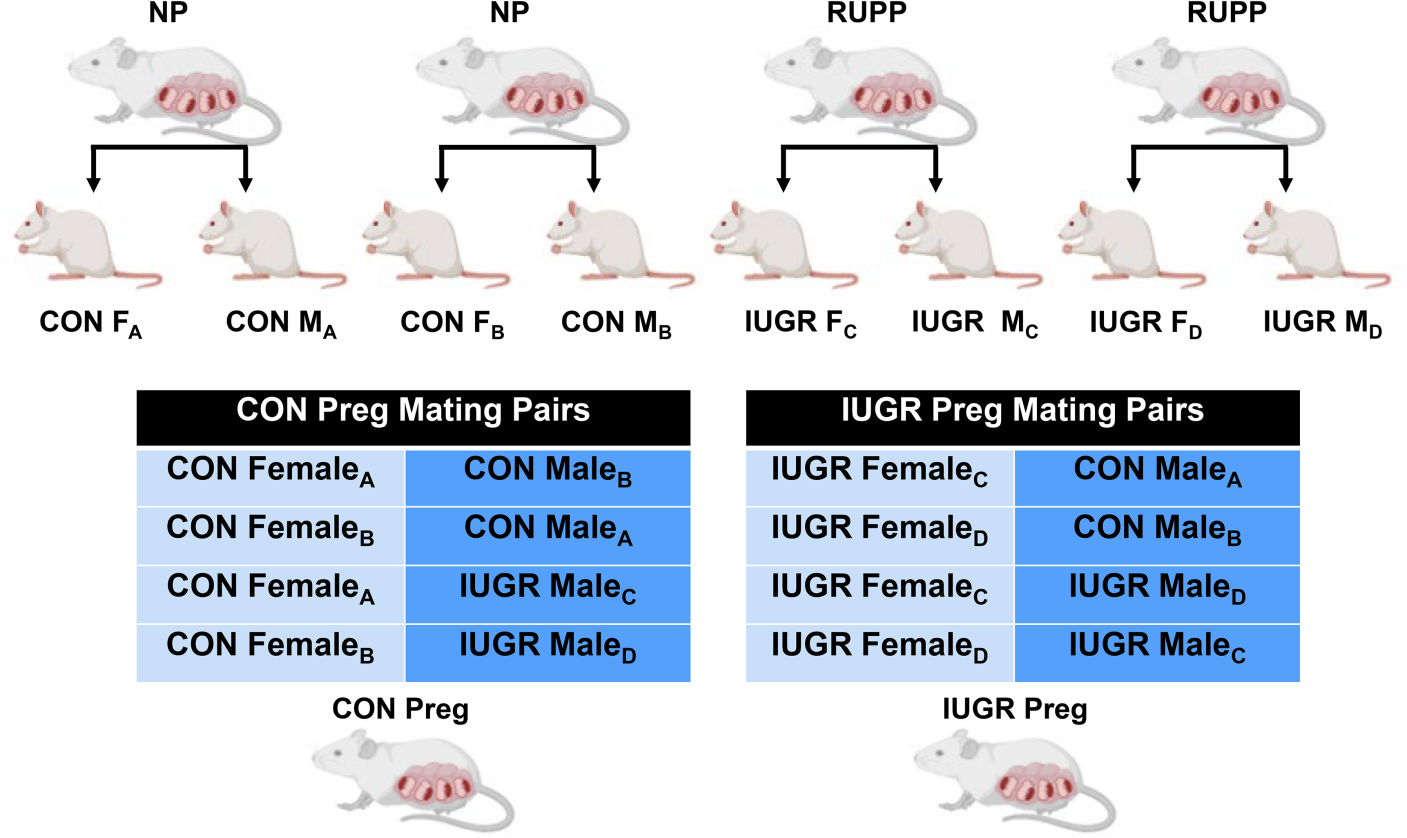
Provides an outline of the experimental design where normal pregnant (NP) rats produce control (CON) offspring. The reduced uterine perfusion pressure (RUPP) rats produce intrauterine growth restriction (IUGR) offspring. The offspring from different dams were then mated according to the mating pair chart generating pregnant CON (CON Preg) and pregnant IUGR (IUGR Preg) rats. To create these pregnancies and avoid litter‐related confounders, we did not mate rats from the same litter.

After weaning, IUGR and CON rats were then separated by sex and group. We used 2–3 RUPP and NP (P1) dams to generate our CON F, IUGR F, CON M, and IUGR M (F1) groups with a *n* = 11 per group. Our final F1 groups were composed of 2–3 rats from each dam. At 10–12 weeks of age, the F1 offspring were mated to create two groups: Pregnant IUGR (IUGR Preg; *n* = 11) and control pregnant (CON Preg; *n* = 11) rats. Pregnant IUGR rats were produced by mating IUGR females to CON males and IUGR females to IUGR males. Whereas the pregnant CON rats were produced by mating CON females to CON males and CON females to IUGR males. Note that no rats from the same litter were mated with each other. Blood pressure (BP), body, organ (placenta, heart, kidney, and brain), and pup weights were recorded on gestational day 19 (late pregnancy, where most of the pathology of PE occurs in patients). Tissue and plasma samples were stored at −80°C to evaluate systemic and local levels of NO bioavailability and OS, using colorimetric biochemical assays and Western blots. See Figure 1 for our experimental design.

### 2.3 Mean arterial pressure (MAP)

MAP measurements were performed on gestational day 19, the day after carotid catheterization, as described by us previously (Booz et al., 2021; Cottrell et al., 2021; Cunningham et al., 2020; Cunningham Jr. et al., 2019; Duncan et al., 2020; Elfarra et al., 2017; Faulkner et al., 2017). BP measurements for female nonpregnant rats at 11–12 weeks of age from normal pregnant (CON F) and RUPP (IUGR F) dams were also recorded using carotid catheterization (Figure S1).

### 2.4 Plasma, organ collection, and storage

Pregnant CON and IUGR rats were euthanized on gestational day 19, in which the blood and organs (kidney, brain, and placenta) were weighed, collected, and stored at −80°C. The kidney cortex and brain were later homogenized in 1:10 lysis buffer for Western blots or a 1X PBS or assay‐specific buffer (50 mg: 500 μL buffer) for assay usage. The homogenate was then centrifuged at 10,000 ***g*** for 15 min to obtain the supernatants. Protein amounts of all supernatants were determined by bicinchoninic acid (BCA) protein assays and used to normalize all experimental values. Note that in the following results, there are differences in *n*‐values. These differences were due to sample depletion during the optimization process.

### 2.5 Nitric oxide (NO) bioavailability

NO bioavailability was evaluated in the plasma, kidney, and brains of pregnant CON and IUGR rats using the nitrate and nitrite colorimetric assay and the protein abundance of neuronal nitric oxide synthase (nNOS), endothelial NOS (eNOS), and phosphorylated endothelial NOS (peNOS) via Western blot analysis. To evaluate the amount of systemic and local amounts of NO, we measured the nitrate and nitrite, which are metabolites of NO, by using the R&D Systems Nitrate/Nitrite Assay Kit (780001, Cayman Chemical, Ann Arbor, MI) as described previously and according to the manufacturer's instructions (Cunningham Jr. et al., 2018). Optical Density (O.D.) measurements were read at 540 nm and corrected at 690 nm, to calculate total NOx in plasma (μM) and organ (μM/μg protein). All organ NOx amounts were normalized to total protein using a BCA assay.

### 2.6 Oxidative stress profile measurements

To evaluate systemic and localized levels of OS, we examined the plasma, kidney, and brain samples of pregnant CON and IUGR rats, measuring 8‐isoprostanes, hydrogen peroxide (H_2_O_2_), and antioxidant capacity levels via biochemical assays as described by us previously (Cunningham Jr. et al., 2013). Plasma 8‐isoprostane, markers of ROS via phospholipid oxidation, were measured by an 8‐Isoprostane ELISA Kit (516351, Cayman Chemical, Ann Arbor, MI). 8‐isoprostanes amounts were calculated in accordance with the manufacturer's instructions and expressed as pg/mL. H_2_O_2_ levels were measured in the kidney and brain per manufacturer's instructions. The final amount of H_2_O_2_ levels were determined via absorbance (570 nm) and normalized to total protein (nMol/mg of protein), for tissue amounts. The antioxidant capacity of plasma and tissue homogenates as described by the manufacturer's instructions was determined by using the antioxidant capacity kit (709001, Cayman Chemical, Ann Arbor, MI) (Cunningham Jr. et al., 2013). Final calculations of antioxidant capacity in plasma were expressed as μM of Trolox and in the kidney and brain as μM trolox/mg protein.

#### 2.6.1 Western blot analysis for NO bioavailability and oxidative stress

Western blots were used to identify and quantify specific proteins in the kidney and brain pertaining to NO bioavailability (peNOS, nNOS, and eNOS) and OS (Cu/ZnSOD, MnSOD, and ecSOD). To ensure protein transfer, normalization, and quantification of total protein amounts in each lane, the ChemiDoc applies UV rays to activate the binding of fluorescent proteins to tryptophan residues, which are quantified by measuring total lane protein intensity. Normally housekeeping proteins like B‐actin or GAPDH are used as controls to assume equal protein loading. However, since housekeeping proteins vary in pregnancy, we utilize the stain‐free normalization process with assistance of the Bio‐Rad ChemiDoc.

Plasma 8‐isoprostane, markers of ROS via phospholipid oxidation, were measured by an 8‐Isoprostane ELISA Kit (516351, Cayman Chemical, Ann Arbor, MI). 8‐isoprostanes amounts were calculated in accordance with the manufacturer's instructions and expressed as pg/mL. H_2_O_2_ levels were measured in the kidney and brain per manufacturer's instructions. The final amount of H_2_O_2_ levels were determined via absorbance (570 nm) and normalized to total protein (nMol/mg of protein), for tissue amounts. The antioxidant capacity of plasma and tissue homogenates as described by the manufacturer's instructions was determined by using the antioxidant capacity kit (709001, Cayman Chemical, Ann Arbor, MI) (Cunningham Jr. et al., 2013). Final calculations of antioxidant capacity in plasma were expressed as μM of Trolox and in the kidney and brain as μM Trolox/mg protein.

More specifically, brain and renal tissues were separately homogenized with 1 mL lysis buffer and centrifuged at 11,000 ***g*** for 20 min. Protein amounts of 100 μg of kidney and brain (organ homogenate, lysis buffer, and 2X Laemmli buffer) were loaded into 4%–20% precast Criterion gels (4568093, Bio‐Rad, Hercules, CA). The protein samples were separated by molecular weight and transferred onto nitrocellulose membranes. Membranes were placed into the Bio‐Rad ChemiDoc MP Imaging System (12003154, Bio‐Rad, Hercules, CA) to ensure protein transfer and determine total protein amounts in each lane. Membranes were blocked with EveryBlot Blocking Buffer (12010020, Bio‐Rad, Hercules, CA) at room temperature. Membranes were blotted with primary antibodies (peNOS, nNOS, eNOS, Cu/ZnSOD, MnSOD, and ecSOD) and incubated overnight at 4°C, on a shaker. Membranes were washed and the appropriate secondary antibodies (Table S1) were added to identify proteins of interest. Membranes were washed again and developed via fluorescents using the Bio‐Rad ChemiDoc MP Imaging System. Results were normalized to total protein amounts per lane (as derived from densitometry analysis using the ImageLab blot image); mean values for CON Preg were set at 100% for comparisons between groups (IUGR vs. CON Preg). Refer to Table S1 for further details of Western blot protocols and Figure S3 for images of the entire membrane, with the ladder and protein of interest, along with membrane total protein amounts.

### 2.7 Sample utilization: Rigor and reproducibility

In this study, no animals were excluded, and all followed the protocol/flowchart in Figure 1. The P1 generation, a subgroup of rats, received the RUPP procedure. All RUPP moms received surgery on the same day (gestational day 14) and had similar physical outcomes as reported by us in previous studies (Cunningham et al., 2020). IUGR offspring were derived from RUPP pregnant rats and CON offspring were derived from normal pregnant rats. In the following results, there are differences in *n*‐values. These differences are due to sample depletion during the optimization process and performing experiments. No rats were mated with their siblings to achieve the experimental or control groups. All parameters of the pregnant offspring are in Table 1. Also all pictures of the entire membrane, with the ladder and protein of interest, along with the membrane total protein amounts are available in Figure S3.

**TABLE 1:** Maternal changes in blood pressure, body weight, organ weight, and fetal weight.

	CON Preg (*n* = 11)	IUGR Preg (*n* = 11)
Blood pressure (mmHg)	100 ± 3	113 ± 4 (*p* = 0.02)
GD 19 body weight (g)	350.3 ± 10.8	330.1 ± 5.2
Heart weight (g/kg BW)	2.5 ± 0.1	2.6 ± 0.1
Kidney weight (g/kg BW)	5.1 ± 0.2	5.1 ± 0.1
Brain weight (g/kg BW)	5.0 ± 0.2	5.4 ± 0.1 (*p* = 0.02)
Total placental weight (g)	5.9 ± 0.8	6.3 ± 0.4
Average placental weight (g)	0.5 ± 0.0	0.6 ± 0.1
Average litter size (# Live)	12.3 ± 1.3	13.6 ± 0.7
Total pup weight (g)	21.0 ± 3.2	26.4 ± 3.7
Average pup weight (g)	2.1 ± 0.3	1.7 ± 0.1 (*p* = 0.05)

*Note*: Displays maternal changes in blood pressure (BP), body weight (grams), heart weight (g/kg body weight), kidney weight (g/kg body weight), brain weight (g/kg body weight), average litter size (# live), total placental weight (g), average placental weight (g), total pup weight (g), and average pup weight (g) at gestational day 19 of pregnant IUGR and CON rats.

### 2.8 Statistical analysis

All data measurements are expressed as mean ± SEM. For comparing BP in groups (CON female × CON male, CON female × IUGR male, IUGR female × CON male, and IUGR female × IUGR male) using a two‐way ANOVA with Bonferroni post hoc test. For all other comparisons of IUGR versus CON Preg, we used the unpaired two‐tailed Student *t*‐test. Statistical significance was defined as a value of *p < 0.05. Note, all the data that support the findings of this study are available upon reasonable request from the corresponding author.

## 3 RESULTS

### 3.1 Maternal blood pressure, body weight, organ weight, and fetal weight

**FIGURE 2:**
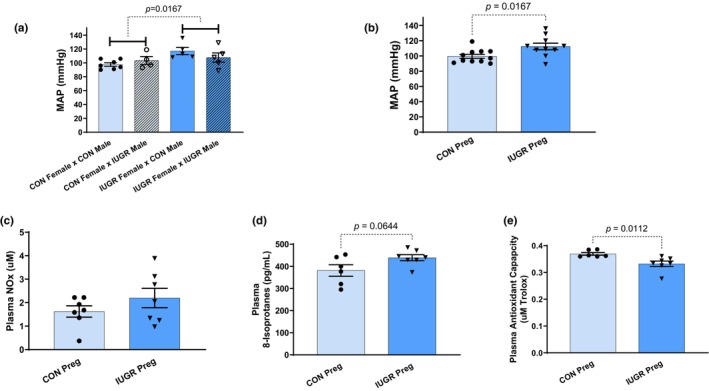
(a) Compares blood pressure (BP) of female CON and IUGR rats mated with male CON and IUGR rats on gestational day 19 of their pregnancies. This figure is composed of four groups: CON female × CON male, CON female × IUGR male, IUGR female × CON male, and IUGR female × IUGR male showing the influence of the mating pair. (b) Examines mean arterial pressure (MAP) between pregnant IUGR (IUGR Preg) and CON (CON Preg) rats. (c–e) Compares plasma NOx, 8‐isoprostanes (marker of systemic ROS), and antioxidant capacity respectively in pregnant IUGR versus CON rats.

There was no difference in MAP when CON females were mated to CON or IUGR males (CON Preg) (Figure 2a). The same results were also observed in IUGR females that were mated with CON or IUGR males (IUGR Preg) (Figure 2a). However, MAP was elevated in IUGR versus CON Preg rats, regardless of male birth status (113 ± 4 vs. 100 ± 3 mmHg, *p* = 0.02) (Table 1; Figure 2b). In other words, IUGR or CON males that were mated with female rats, appeared to have no influence on maternal BP. Because there was no difference in BP with male birth status, the groups were organized based upon female birth status, in which we found a statistical increase (~13 mmHg) in BP between IUGR vs. CON Preg. Additionally, there was no difference in BP between IUGR and CON rats in the absence of pregnancy (Figure S1). This indicates that the increase in BP from pregnant IUGR rats versus pregnant CON rats was not due to the elevation of BP before pregnancy, but rather during their pregnancy. There was no change in gestational day 19 body weight, heart weight, kidney weight, average litter size, total placental weight, average placental weight, and total pup weight (Table 1). However, there was a significant 9% increase in total brain weight in pregnant IUGR rats (5.4 ± 0.1 vs. 5.0 ± 0.2 g/kg BW, *p* = 0.02) (Table 1). Although not significant, there was a ~20% trending reduction in the average pup weight of pregnant IUGR rats (1.7 ± 0.1 vs. 2.1 ± 0.3 g/kg BW, *p* = 0.05) (Table 1).

### 3.2 Systemic and local evaluation of NO bioavailability and oxidative stress

#### 3.2.1 Plasma

There were no changes in plasma NOx levels in pregnant IUGR rats (2.2 ± 0.4 vs. 1.6 ± 0.2 μM, NS) (Figure 2c). Plasma 8‐isoprostane (marker for ROS) levels trended upward in pregnant IUGR rats (381.3 ± 26.1 vs. 439.2 ± 13.6 pg/mL, *p* = 0.06) (Figure 2d). Plasma antioxidant capacity was lower in pregnant IUGR rats (0.3 ± 0.0 vs. 0.4 ± 0.0 μM Trolox, *p* = 0.01) (Figure 2e).

#### 3.2.2 Kidney

**FIGURE 3:**
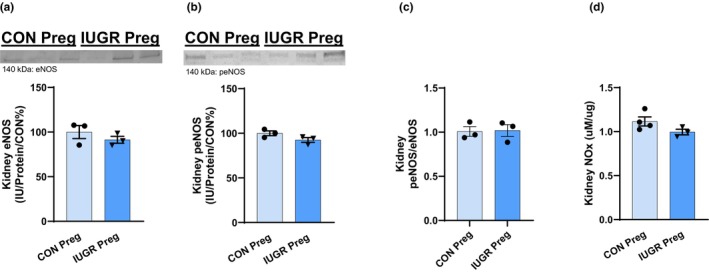
Kidney cortex NO bioavailability was evaluated by examining (a) eNOS, (b) peNOS, (c) eNOS/peNOS, and (d) NOx concentrations in pregnant IUGR versus CON rats.

**FIGURE 4:**
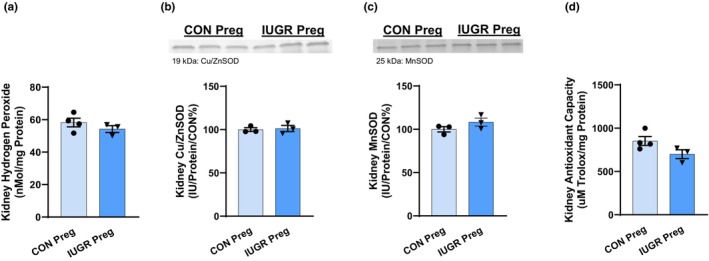
Kidney cortex (a) H_2_O_2_, (b) Cu/ZnSOD, (c) MnSOD, and (d) antioxidant capacity of IUGR and CON pregnant rats.

To measure kidney NO bioavailability, we examined the protein levels of endothelial NOS (eNOS) and phosphorylated endothelial NOS (peNOS) (the enzymes that generate NO production), along with NOx (nitrate and nitrite, metabolites of NO) production. No changes were observed in eNOS, peNOS, the ratio of eNOS/peNOS protein abundance, and NOx levels with pregnant IUGR rats (Figure 3a–d). OS was evaluated in the kidney cortex by examining H_2_O_2_ (marker of ROS) levels, copper/zinc superoxide dismutase (Cu/ZnSOD; cytosolic SOD, antioxidant), manganese superoxide dismutase (MnSOD; mitochondrial antioxidant), and antioxidant capacity. No significant changes were observed in H_2_O_2_ levels, Cu/ZnSOD and MnSOD protein abundances, and antioxidant capacity (Figure 4a–d).

#### 3.2.3 Brain

**FIGURE 5:**
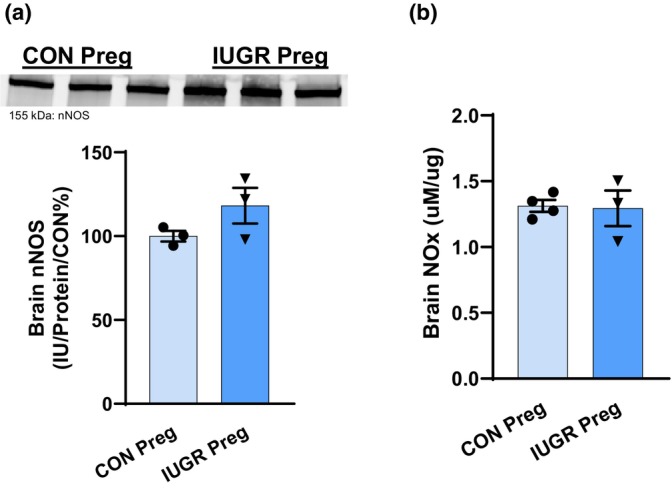
Brain NO bioavailability was evaluated by examining (a) nNOS and (b) NOx (nitrate and nitrite) levels in pregnant IUGR versus CON rats.

**FIGURE 6:**
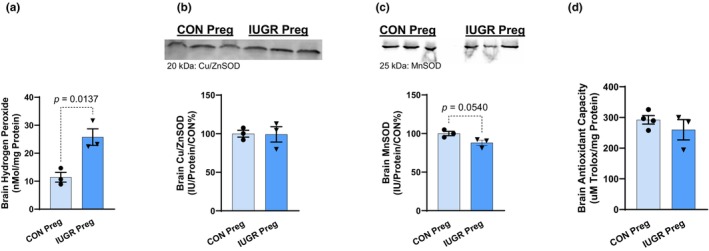
Brain (a) H_2_O_2_, (b) Cu/ZnSOD, (c) MnSOD, is and (d) antioxidant capacity of pregnant IUGR and CON rats.

No changes in brain neuronal NOS (nNOS) protein abundance and NOx were observed in pregnant IUGR rats (Figure 5a,b). However, H_2_O_2_ (marker of ROS) concentration was drastically increased, ~2.4‐fold, in pregnant IUGR rats (25.8 ± 5.1 vs. 11.4 ± 3.0 nMol/mg protein, *p* = 0.01) (Figure 6a). There was no change in brain Cu/ZnSOD (Figure 6b), but there was a downward trend in brain MnSOD in pregnant IUGR rats (88 ± 3 vs. 100 ± 3 IU/Protein/CON%, *p* = 0.05) (Figure 6c). There was no difference in brain antioxidant capacity in pregnant IUGR rats (260 ± 33 vs. 292 ± 14 μM Trolox/mg protein, NS) (Figure 6d).

## 4 DISCUSSION

Pregnant IUGR rats, born from hypertensive placental ischemic (RUPP) dams with surgically induced PE, have HTN and smaller pups at late gestation (gestational day 19). In addition, they have elevated systemic and cerebral OS as well as larger brain size (Warrington et al., 2015), which may lead to cerebral damage and/or injury commonly observed in preeclamptic women with cerebral edema. Despite the cerebral damage that may be associated with this model, the kidneys appear to be less damaged or protected, by showing no differences in NO bioavailability and OS. Therefore, more observations are needed to determine if there are dysfunctional changes in the renal vasculature, structure, and function. Whether or not HTN in these pregnant IUGR rats is due to cerebral damage and/or the cerebral OS in this model, is not yet known and will be the focus of future studies. However, pregnant IUGR rats from hypertensive placental ischemic rat dams, in this study, show symptoms of a preeclamptic‐like phenotype, such as increased BP, low birth weight offspring, and increased OS. Thus, suggesting a new model to study PE.

PE is hypothesized to be caused by abnormal trophoblast invasion in the mother's endometrium during pregnancy (LaMarca et al., 2016). The decrease in trophoblast invasion causes abnormal placental spiral artery remodeling during placentation that results in a deficit of oxygen and nutrient exchange between the mother and the fetus. The net result of these abnormalities causes placental ischemia, endothelial dysfunction, and OS (Amaral et al., 2017).

Both the mom and fetus have short‐ and long‐term health risks associated with the PE (Amaral et al., 2017; Cunningham Jr. & LaMarca, 2018; Turbeville & Sasser, 2020). Furthermore, daughters of women with PE, have increased risk of having PE themselves (Sherf et al., 2019). In 2001, a genetic study performed in Utah found that PE is influenced by both maternal and paternal genes. However, the maternal influence was greater (Esplin et al., 2001). Our studies mirror these findings and illustrate that birthing status (IUGR or CON) of the pregnant dam (mother) was a greater predictor of HTN during pregnancy (the major requirement for PE), regardless of the paternal birthing status (IUGR vs. CON) (Cnattingius et al., 2004; Esplin et al., 2001; Galaviz‐Hernandez et al., 2018). In Skjaerven et al., daughters born of a preeclamptic pregnancy exhibit a two‐fold greater risk of developing PE themselves, thus emphasizing this increased risk of PE on daughters that were born from preeclamptic mothers (Skjaerven et al., 2005). This finding has also been found in other population studies, showing a greater influence of maternal versus paternal genetic influence on offspring having PE (Cnattingius et al., 2004; Esplin et al., 2001; Galaviz‐Hernandez et al., 2018). Furthermore, in a more recent study, it was concluded that if the mother was an IUGR baby or had PE, then daughters had a higher incidence of PE in their pregnancies (Sherf et al., 2019). In summary, our data along with data in the literature from rodents and human studies, show that pregnant IUGR offspring display preeclamptic‐like symptoms during pregnancy (Gallo, Tran, Moritz, Jefferies, & Wlodek, 2012; Gallo, Tran, Moritz, Mazzuca, et al., 2012; Paauw et al., 2017; Zetterstrom et al., 2007).

Few studies have examined pregnant rodents from second‐generation preeclamptic pregnancies (Gallo, Tran, Moritz, Jefferies, & Wlodek, 2012; Gallo, Tran, Moritz, Mazzuca, et al., 2012; Paauw et al., 2017). Gallo et al. have shown that aged 12‐month Wistar Kyoto restricted (IUGR) female rats from placental ischemic dams have elevated BP, before and during pregnancy; with normal renal and metabolic pregnancy adaptations. However, these aged, restricted rats display a normal drop in BP during pregnancy (Gallo, Tran, Moritz, Jefferies, & Wlodek, 2012). In addition, when they observed younger 4‐month nonpregnant and pregnant restricted rats, they saw no differences in BP between nonpregnant and pregnant restricted and CON rats. Similarly, our IUGR 2.5‐month Sprague Dawley rats from placental ischemic dams have no difference in BP before pregnancy in comparison to nonpregnant CON rats (Figure S1). In other words, our nonpregnant IUGR rats have the same BP as the nonpregnant CON rats at the same age, thus showing IUGR female rats do not have HTN before pregnancy. Moreover, our pregnant IUGR rats have an elevated BP of ~13 mmHg higher than pregnant CON rats (Figure 2B). Additionally, our pregnant IUGR rats in this study do not exhibit the normal drop in BP. However, the pregnant CON rats show the expected decrease in BP during pregnancy, as shown in many clinical and human studies of pregnancy (Alexander et al., 2001; Ayala et al., 1997; Cunningham Jr. et al., 2015; Lindheimer et al., 1976; Morris et al., 1996; Sladek et al., 1997).

Although there are several other models of PE, they are all derived from either genetic and selective breeding (BPH mice and Dahl salt sensitive rat models), pharmacological (sFlt‐1, AT‐1‐AA, L‐NAME, and LPS models), and/or surgical interventions (RUPP model) (Bakrania et al., 2022; Campbell et al., 2018; Gatford et al., 2020; Granger et al., 2006; LaMarca et al., 2016; Taylor & George, 2022). Note that the soluble FMS‐like tyrosine kinase 1 (sFlt‐1) model, angiotensin 2 type 1 receptor autoantibody (AT1‐AA) model, RUPP, L‐Nitro‐arginine methyl ester (L‐NAME), and low‐dose lipopolysaccharide (LPS) models are all animal models that express HTN, produce IUGR offspring, have decreased NO bioavailability, mitochondrial dysfunction, and elevated levels of OS, inflammation, and antiangiogenic factors (Turbeville & Sasser, 2020). Whereas the inflammatory models of PE, such as tumor necrosis factor α (TNF‐α) and interleukin 17 (IL‐17) contain all previous characteristics, but varied results in producing IUGR offspring (LaMarca et al., 2016; Taylor & George, 2022). The BPH mouse and Dahl salt sensitive (SS) rat models produce HTN, IUGR offspring, and elevated levels of OS, inflammation, and antiangiogenic factors. Although the BPH and Dahl SS rodent models have provided a multitude of information on the pathology of PE, these models still require intervention via selective breeding. Both the BPH mouse and Dahl salt sensitive (SS) rat models were derived from selective breeding protocols to select the specific subtypes of HTN in rodents. These hypertensive rodents were then selected and mated to generate the desired hypertensive phenotype of the rodents before and/or during pregnancy. Moreover, these models also produce rodents with metabolic dysfunction, obesity, increased adiposity, and/or borderline HTN before pregnancy (Sones et al., 2021). In the Dahl SS rat model, when a high‐salt diet is administered, the nonpregnant Dahl SS rat experiences an increase in BP, which continues to rise, during pregnancy. Thus, one may conclude that the HTN and preeclamptic‐like phenotype in these rodent models are a continuation of an increase in BP from the nonpregnancy state, that is, models of chronic HTN with superimposed PE. However, this is not the case with our rats, as shown by IUGR rat offspring that have no differences in BP without pregnancy (Figure S1). Thus, our IUGR female rats are normotensive, expressing no difference in BP in comparison to CON rats without pregnancy. This observation has been investigated in other studies, such as by Alexander et al, that show IUGR female rats derived from the placental ischemic RUPP model of PE, after puberty (after 8 weeks of age), do not have elevated BP compared to female CON rats (Alexander et al., 2015; LaMarca et al., 2016). However, the male IUGR rats have increased BP, and these sex differences may be due to the role of estrogen (Davis et al., 2019).

It is important to note that our nonpregnant female IUGR rats at 11–12 weeks of age did not have an increase in BP, changes in circulating glucose levels, nor changes in body weight compared to nonpregnant female CON rats (Figures S1 and S2). Note that we did not measure our pup weight of these IUGR offspring (F1 rats) before 4 weeks of weaning for the fear of maternal rejection and rough handling of the offspring. Conversely, after the third week of weaning was completed, the offspring were closely observed for changes in weight, metabolic measurements (such as glucose), and BP until 11–12 weeks of age (Figures S1 and S2). Thus, based on our data, it is reasonably assumed that our female IUGR rats did not have chronic HTN before pregnancy, but rather developed preeclamptic‐like characteristics, such as HTN, during pregnancy.

Renal damage, as seen by proteinuria, elevated OS, and low NO bioavailability are common markers in preeclamptic animal models and humans (Amaral, Cunningham Jr. et al., 2015; Bakrania et al., 2022; Eiland et al., 2012; Lowe, 2000). However, PE is a syndrome with a variety of possible symptoms and organ dysfunctions. For a pregnancy to be classified as preeclamptic, renal damage is not required. As of 2013, proteinuria, although a helpful diagnostic tool, does not alone reflect the severity or presence of PE in accordance with the American College of Obstetricians and Gynecologists (Erez et al., 2022; Fishel Bartal et al., 2022; Homer et al., 2008; Karrar & Hong, 2023; Reddy & Jim, 2019). In our study, HTN and cerebral OS were present in pregnant IUGR rats, but the kidneys appeared to be less damaged or protected, as observed by no changes in renal cortex NO bioavailability and OS. The kidney cortex was explicitly used in our studies since NO‐mediated renal hemodynamic changes, which influence glomerular filtration rate (GFR), occur within the cortex. During a normal pregnancy, NO levels increase and cause a decrease in renal vascular resistance (RVR). This decrease in RVR will cause vasodilation of both afferent and efferent arterioles to increase renal blood flow and GFR. However, in preeclamptic states, NO is lowered, increasing RVR, resulting in a reduction of blood flow and GFR (Amaral, Cunningham Jr. et al., 2015; Conrad & Davison, 2014; Hussein & Lafayette, 2014; Jeyabalan et al., 2003). Furthermore, other preeclamptic animal studies show a decrease in renal cortex eNOS amounts and NO metabolites (Cunningham Jr. et al., 2013; Wei et al., 2021).

Perhaps the reason why this model of PE shows no decrease in renal NO bioavailability due to an increase and/or no loss in antioxidant capacity at this age. Nevertheless, more tests are needed to confirm this response. The kidneys, major regulators of BP, in the pregnant IUGR rat may not play a prominent role in the pathophysiology of HTN and developments of preeclamptic‐like symptoms in our model. Other markers of renal damage such as decreased GFR and increased albumin, proteinuria, creatinine, and vascular tone were not tested in this study, but are needed for further renal function and hemodynamic responses.

An important contributor to BP regulation is the brain. The common occurrence of cerebral symptoms reveals that preeclamptic women are vulnerable to brain damage and abnormalities (Bergman et al., 2019; Cipolla et al., 2018; Cunningham Jr. & LaMarca, 2018; Escudero et al., 2023). The increase in the BP and preeclamptic‐like pathophysiology could be caused or facilitated by cerebral damage. The link between cerebral damage and HTN is unknown in this model and will be addressed in future studies. To determine the mechanisms and development of HTN and cerebral damage, we will conduct timeline and drug manipulation experiments (Reddy & Jim, 2019). Many human PE studies indicate the presence of cerebral OS and edema (Barron et al., 2021; Bergman et al., 2019; Rains et al., 2021). Specifically, our data supports these findings by showing increased H_2_O_2_ concentrations along with a decreasing trend in MnSOD, and increased brain weight. Note that some studies suggest that brain size decreases during a normal pregnancy and PE (Oatridge et al., 2002). In contrast, our study found an increase in brain size due to possible edema that is commonly expressed in preeclamptic women (Manoharan et al., 2024; Warrington et al., 2014). This is like the findings of Warrington et al. that showed an increase in water content in the anterior brain of preeclamptic rats (Warrington et al., 2015). Perhaps the variation in timing and severity of PE, which occurs in different women, may have differing effects on the brain. Moreover, in our studies, we took the absolute brain weights and did not calculate the brain water content. Thus, the increase in brain size, seen in our study, could be due to edema. If we subtract the brain water content, we may see smaller brains. Additional studies and measurements are warranted to make this conclusion. Furthermore, studies show that in the circulating blood of PE women, there are elevated levels of malondialdehyde (MDA), 8‐isoprostane and leukocyte DNA damage, along with lower levels of the antioxidants: glutathione, lycopene, vitamin C, vitamin E, and altered amounts and activity of the antioxidant enzymes (superoxide dismutase, catalase, and glutathione peroxidase). This imbalance between ROS and antioxidants indicates elevated OS, which may contribute to neurological dysfunction in the mothers and neurodevelopment disorders in offspring (Barron et al., 2021). Since the brain is implicated in the pathophysiology of PE, perhaps brain‐directed therapies, to increase antioxidant capacity and reduce ROS, could ameliorate the preeclamptic symptoms.

Although this study examines some of the markers associated with PE, there are many more to evaluate in the future. For example, mitochondrial dysfunction, pro‐inflammatory factors/cells (Il‐6 and IL‐17), antiangiogenic factors (sFlt‐1, soluble endoglin), and placental ischemia (Cunningham Jr. et al., 2019; Smith et al., 2021). Placental ischemia is one of the major hallmarks of PE, and unfortunately, we did not have the proper equipment to make these measurements in this study. Moreover, vasculature resistance needs to be evaluated to determine its possible role in the increase in BP displayed in our pregnant IUGR offspring. Studies have shown that female IUGR rodent offspring in the absence of pregnancy have vascular dysfunction (Anderson et al., 2006; Mazzuca et al., 2010). However, whether these IUGR offspring with vascular dysfunction have elevated BP or not is controversial. Some studies show no change in BP in IUGR offspring with only uterine vascular dysfunction (Anderson et al., 2006), while others show mesenteric vascular dysfunction, and BP elevation in both first‐ and second‐generation IUGR offspring (Anderson et al., 2006; Mazzuca et al., 2010). To confirm our results and provide a more comprehensive BP profile, radiotelemetry probes would be ideal. However, because we did not have access to radiotelemetry in this study, we could not perform these experiments.

In this study, we have two major limitations. First, we used multiple female rats from a litter to compose our F1 CON and IUGR Preg offspring groups, which could potentially introduce a litter bias. Although, some offspring came from the same litter, not all rats in each group came from the same litter. Additionally, this is an observational study that does not propose a cause and effect and/or mechanism. Therefore, we cannot determine how one factor affects other downstream factors, such as the how kidney and brain NO bioavailability and oxidative stress influence blood pressure. Moreover, this study is not longitudinal, thus we cannot determine which variable changes first in IUGR Preg offspring.

In conclusion, this study demonstrates that F1 female IUGR rats, born from hypertensive PE (RUPP) dams, have preeclamptic‐like symptoms during pregnancy. These pregnant IUGR rats have HTN, elevated systemic and cerebral OS, enlarged brains, and smaller pups during late gestation, which are all clinical observations of women with PE. Despite the cerebral damage that may be associated with this model, the kidneys appear to be less damaged and/or protected, showing no differences in NO bioavailability and OS (imbalance between ROS and antioxidants). Although there appears to be no drastic changes in the kidney, more observations are needed to determine if there are dysfunctional changes in the renal vasculature structure and function. Whether or not HTN in these pregnant IUGR rats is due to cerebral damage (increase brain size due to edema) and/or the cerebral OS to cause cerebral damage in this model, is not yet known and will be the focus of future studies. Note that the increase in OS in this study is supported by ROS (increasing trend to circulating 8‐isoprostanes and decreased total antioxidant capacity; increased H_2_O_2_, and a decreasing trend in MnSOD in the brain). This imbalance of decreased or stationary antioxidants, and increasing ROS, suggests an overall net increase in OS balance. Changes in cerebral OS observed in pregnant IUGR rats suggest that antioxidant‐specific and/or organ‐targeted therapy may be beneficial to PE women, although more studies are needed to verify this hypothesis.

Findings from this study are important to shift focus towards preventative care, especially in at‐risk populations, in which the mother had PE. Moreover, the use of antioxidant‐specific and organ‐specific therapy could be helpful to reduce the burden of PE, specifically in pregnant women that experienced IUGR in utero. Knowing the pregnancy complications and/or the IUGR status of the pregnant mom, may provide insights for the risk of developing PE during the daughter's pregnancy. Therefore, this study advises physicians to carefully examine populations of women that were born IUGR and/or whose mother was preeclamptic before and during their pregnancy. Thus, we advocate that all pregnant women should know their mother's pregnancy history and/or story, because it may be beneficial to their overall health.

## AUTHOR CONTRIBUTIONS

MC (PI) conceived and designed research, helped to perform experiments, analyzed data, interpreted results of experiments, edited and revised manuscript, and approved final version of manuscript. JS (first author of the manuscript) performed experiments, analyzed data, interpreted results of experiments, prepared figures, drafted manuscript, and edited and revised manuscript. MP, WC, SS, KJ, and AC performed experiments, analyzed data, interpreted results of experiments, edited and revised manuscript. AH assisted with interpretation and editing the manuscript. JS, JE, RT, AS, and BF performed experiments, analyzed data, and interpreted results. GB analyzed data, interpreted results of experiments, prepared figures, and edited and revised manuscript.

## FUNDING INFORMATION

This work was supported by a grant from the American Heart Association to MC (AHA 18CDA34110264). GB was supported in part by the National Institute of General Medical Sciences of the National Institutes of Health under Award Number P20GM121334. The content of this manuscript is solely the responsibility of the authors and does not necessarily represent the official views of the National Institutes of Health.

## CONFLICT OF INTEREST STATEMENT

None of the authors have any conflicts of interest to disclose.

## ETHICS STATEMENT

All animal experiments were conducted in accordance with the guidelines of the Institutional Animal Care and Use Committee (IACUC) at the University of Mississippi Medical Center, and all procedures were approved by the IACUC protocol number 1543. Animals were housed and cared for according to the National Institutes of Health (NIH) Guide for the Care and Use of Laboratory Animals. In addition, the data that support the findings of this study are available from the corresponding author, [MC], upon request.

## Supporting information


Appendix S1.
