# Supplementary material for: Intrauterine growth‐restricted pregnant rats, from placental ischemic dams, display preeclamptic‐like symptoms: A new rat model of preeclampsia
Source: Physiol Rep. 2024 Oct 31;12(21):e70112. doi: 10.14814/phy2.70112 (PMC11527824; doi:10.14814/phy2.70112)
Supplement: Supplementary file 1 — Appendix S1. [file PHY2-12-e70112-s001.docx]

**SUPPLEMENTAL TABLES AND FIGURES**

**Supplemental Table 1**


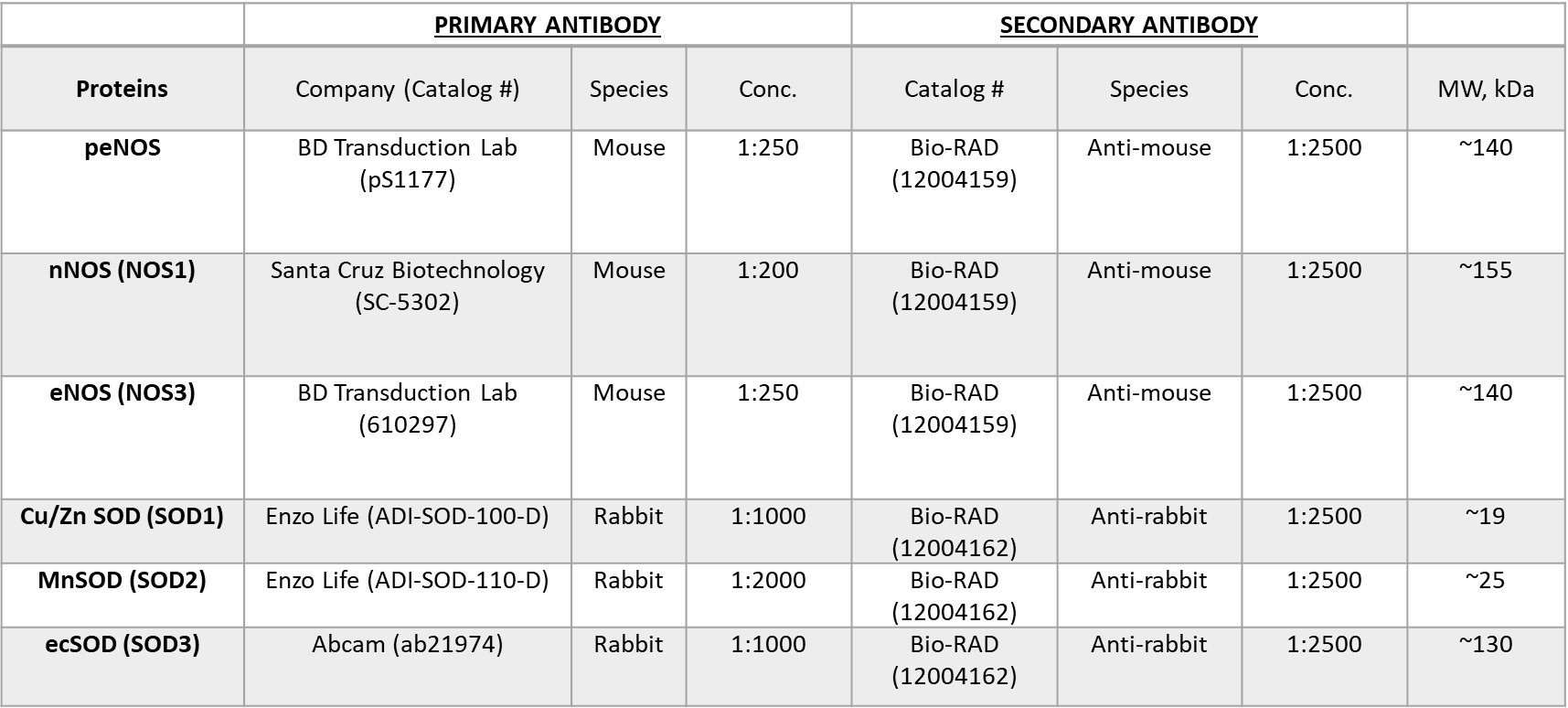


**Supplemental Table 1)** is a comprehensive list of antibodies used in this study along with secondary antibodies used for detection. The company source, catalogue number, and concentrations of antibodies used are displayed.

**Supplemental Figure 1**

**Supplemental Figure 1**) Mean Arterial Pressure (MAP; mmHg) of non-pregnant IUGR (IUGR F) and CON (CON F) rats at 11-12 weeks of age.

**Supplemental Figure 2A**

**Supplemental Figure 2A**) Compares body weight averages (grams) from 4 weeks to 12 weeks of age non-pregnant female IUGR (IUGR F) and CON (CON F) rats.

**Supplemental 2B**

**B)** Fasting glucose averages (mg/dL) of non-pregnant female IUGR (IUGR F) and CON (CON F) rats at 4, 8, and 12 weeks of age.

**Supplemental Figure 3A**


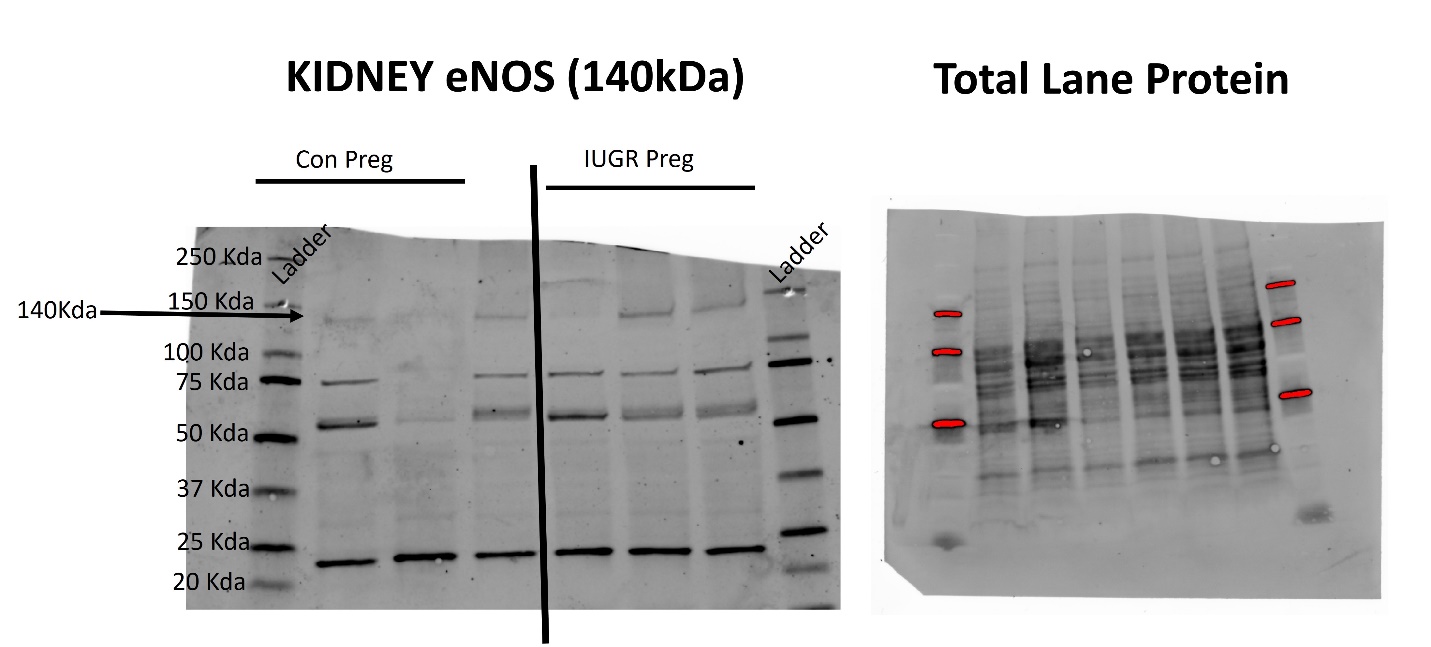


**A)** Kidney cortex eNOS Starbright 700 blot and total lane protein

**Supplemental 3B**


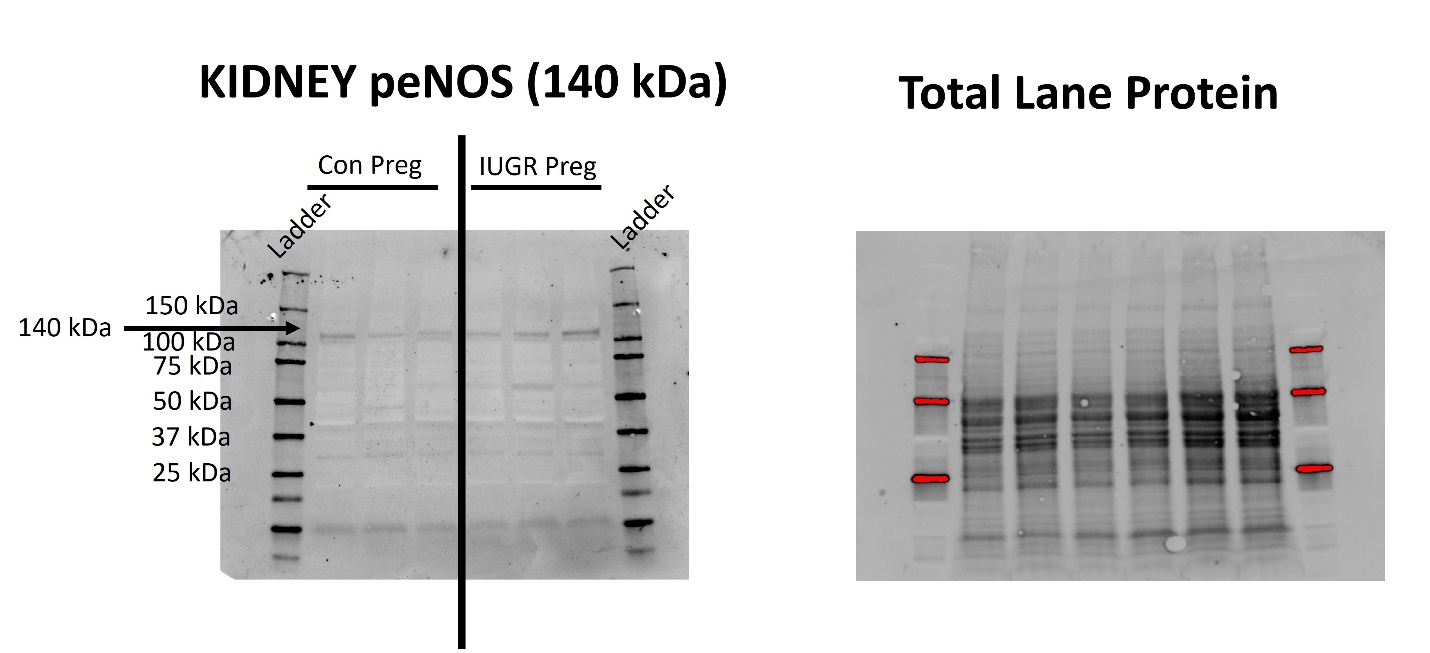


**B)** Kidney cortex peNOS Starbright 700 blot and total lane protein

**Supplemental 3C**


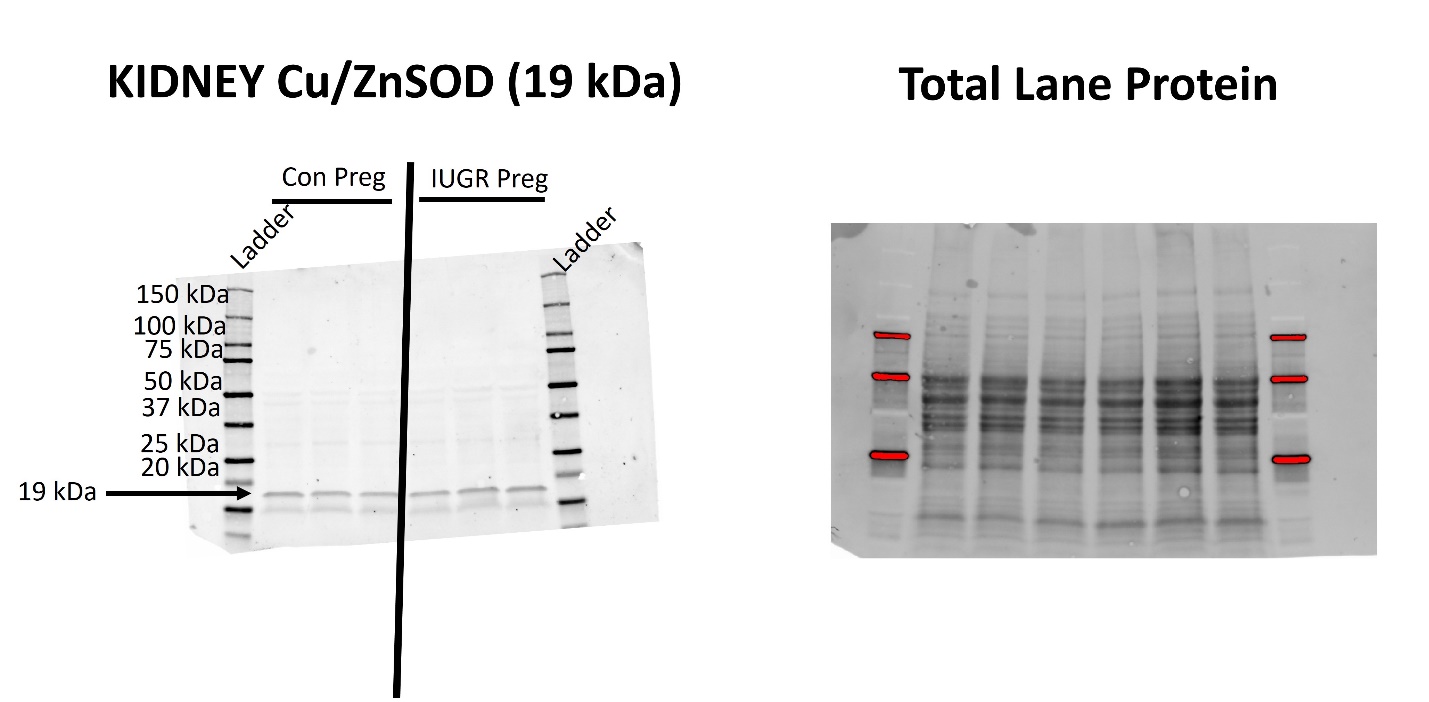


**C)** Kidney cortex Cu/ZnSOD Starbright 700 blot and total lane protein

**Supplemental 3D**


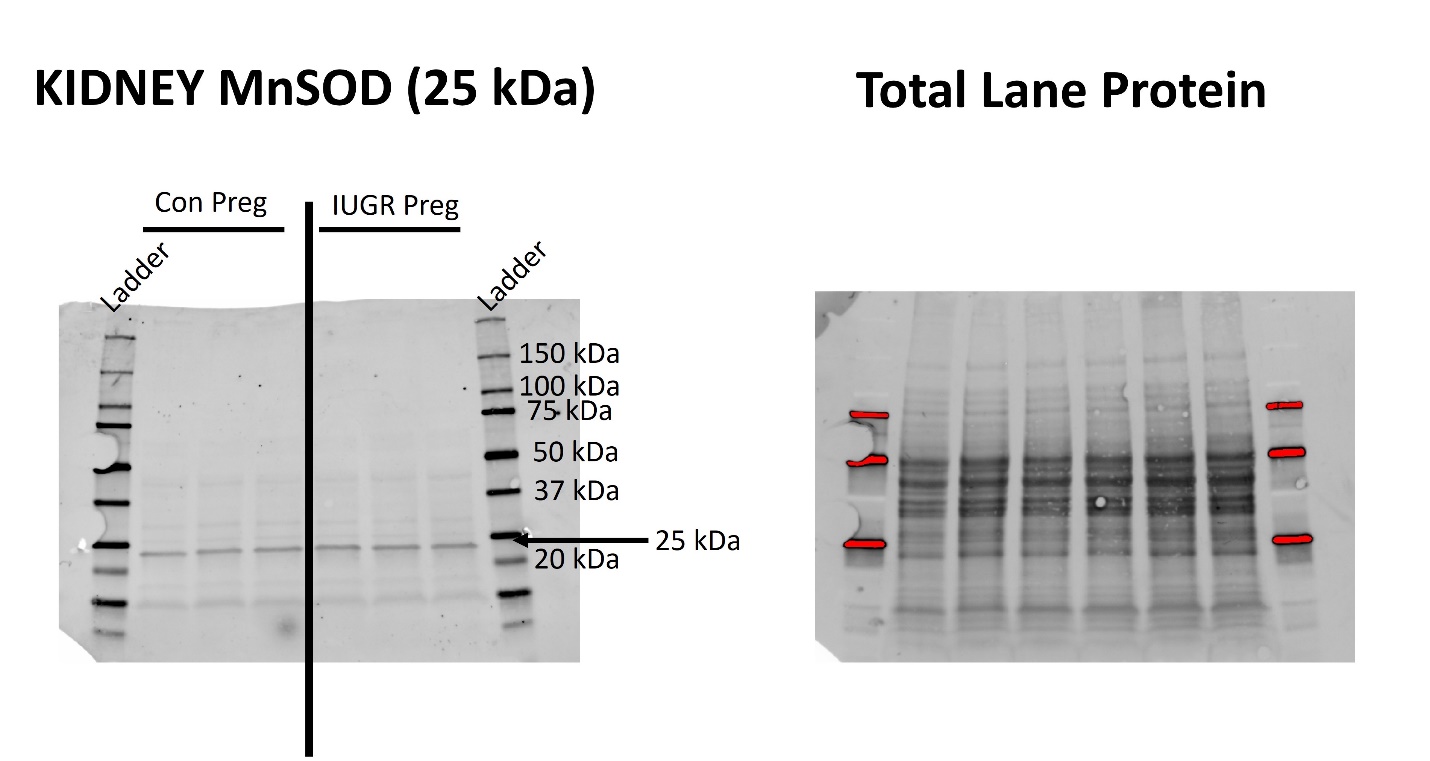


**D)** Kidney cortex MnSOD Starbright 700 blot and total lane protein

**Supplemental 3E**


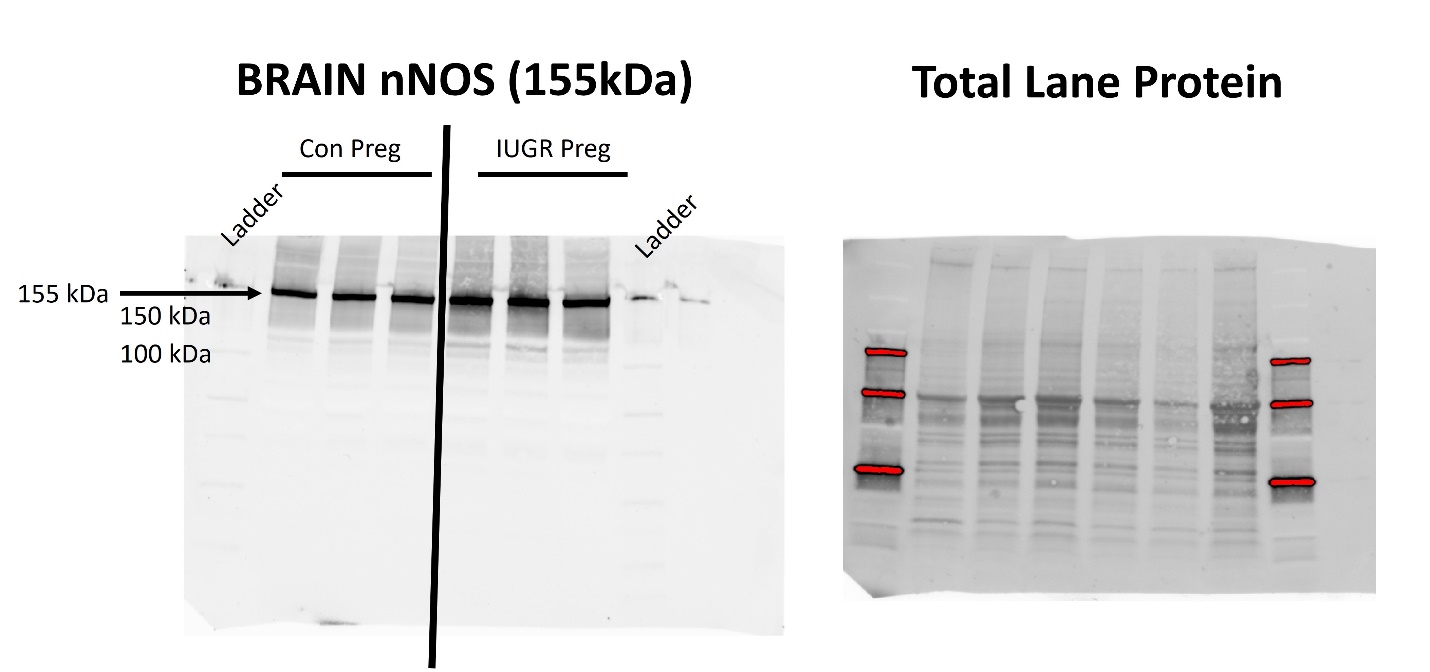


**E)** Brain nNOS Starbright 700 blot and total lane protein

**Supplemental 3F**


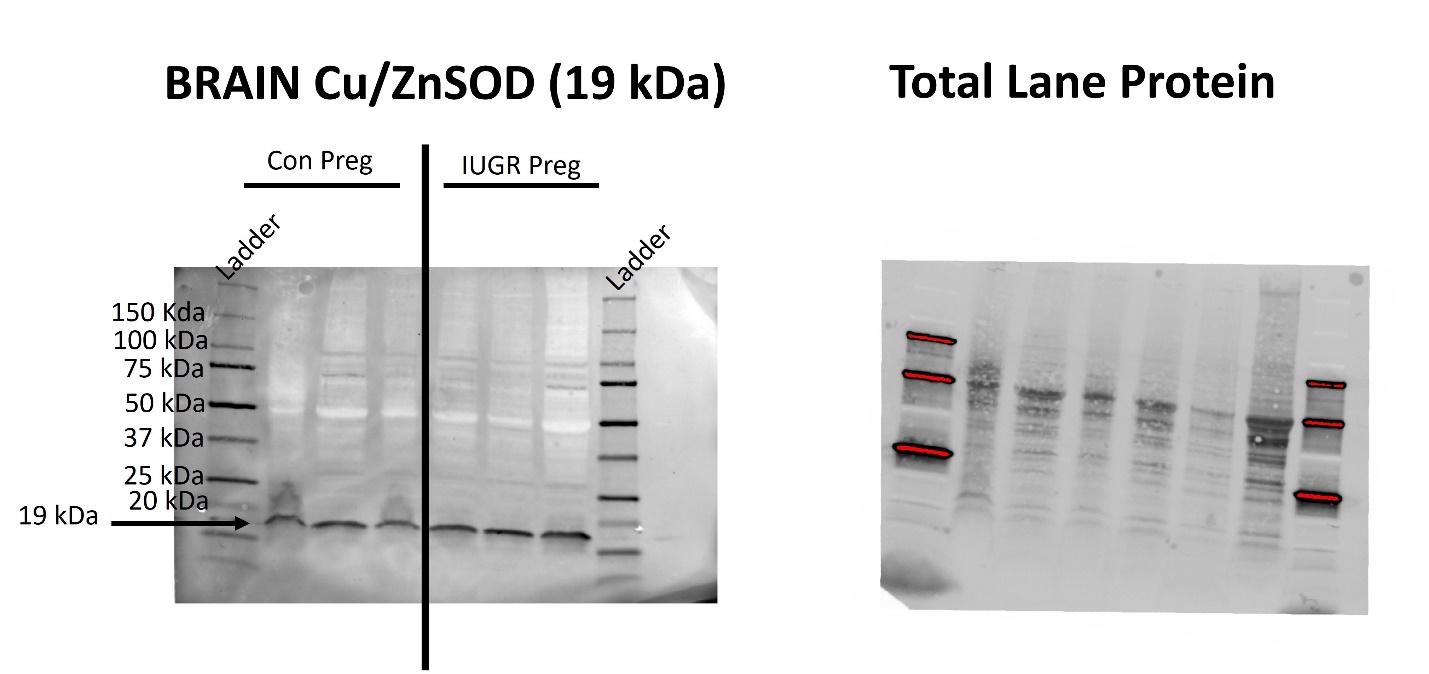


**F)** Brain Cu/ZnSOD Starbright 700 blot and total lane protein

**Supplemental 3G**


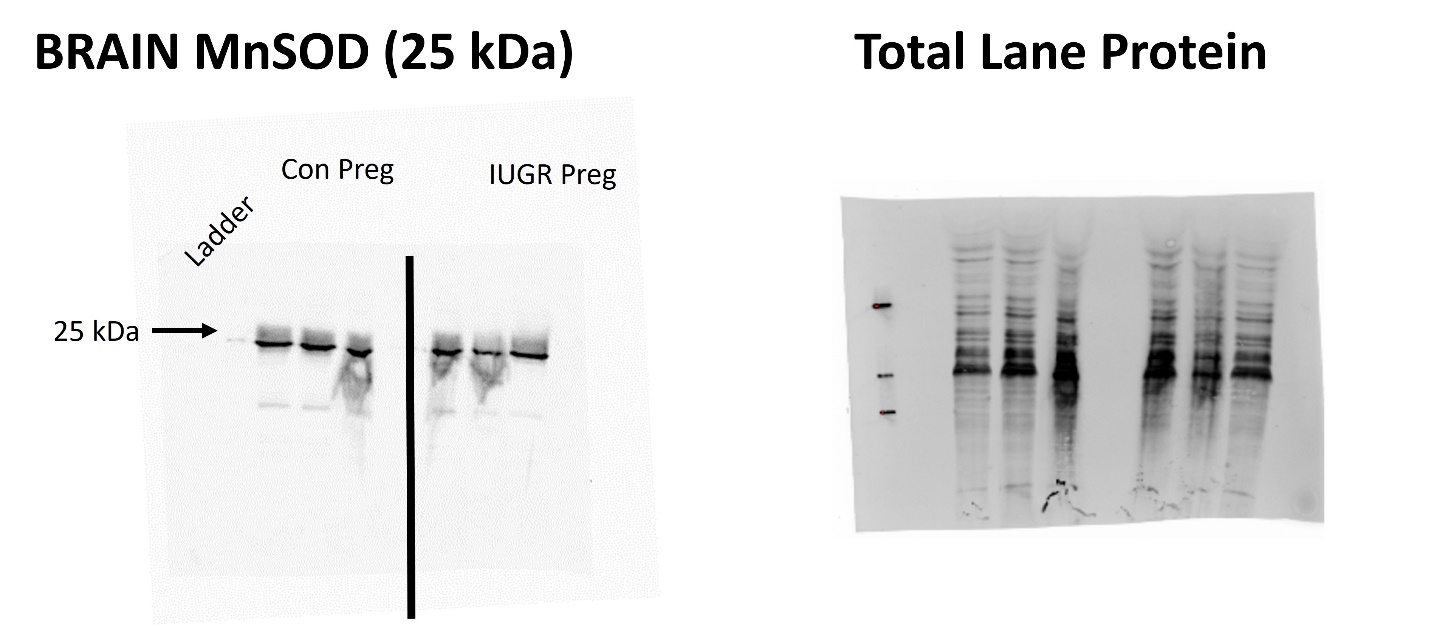


**G)** Brain MnSOD Starbright 700 blot and total lane protein
